# Functional Sensorimotor‐Based Training for Balance and Gait Enhancement in Sedentary Older Women: A Randomized Clinical Trial

**DOI:** 10.1002/hsr2.72115

**Published:** 2026-04-26

**Authors:** Parisa Sedaghati, Fatemehzahra Talebi kenari

**Affiliations:** ^1^ Department of Sport Injuries and Corrective Exercise, Faculty of Sport Sciences University of Guilan Rasht Iran

**Keywords:** elderly rehabilitation, fall risk, functional mobility, motor performance, proprioception

## Abstract

**Background and Aims:**

Sensorimotor training has emerged as a promising intervention to enhance gait and balance and potentially reduce fall risk. However, evidence on its effectiveness and the short‐term durability of these effects is limited, especially in sedentary older women. This study therefore aimed to investigate the impact of functional sensorimotor‐based training on balance, gait performance, and fall risk in this population.

**Methods:**

This randomized controlled clinical trial investigated the effects of sensorimotor training on balance and gait in sedentary older women. Thirty participants aged 60–75 years residing in nursing homes in Guilan province were randomly assigned to an experimental group (*n* = 15) or a control group (*n* = 15). Assessments were conducted at pre‐test, post‐test (after 8 weeks), and follow‐up, measuring balance, gait parameters, joint position sense, and lower‐limb function. The experimental group completed a 50‐min functional sensorimotor‐based training program, three times per week for 8 weeks, while the control group engaged only in general physical education activities. Data were analyzed using RMANOVA, followed by Bonferroni post hoc tests, with significance set at *p* < 0.01. Analyses were performed using SPSS version 25.

**Results:**

The results showed that there was a significant difference between the experimental and control groups in variables of the Fear of falling (F = 9.066; *p* = 0.001; η² = 0.40), Time Up & Go (F = 23.495; *p* = 0.01; η² = 0.45), Time of Walking (F = 89.42; *p* = 0.001; η² = 0.85), and 5Sit& up (F = 22.118; *p* = 0.001; η² = 0.62). In the experimental group, improvements in balance, gait, joint position sense, and lower‐limb function were partially retained at follow‐up. Compared with pre‐test values, changes in FOF (*p* = 0.012, d = 0.28), TUG (*p* = 0.001, d = 1.22), 10MWT speed (*p* = 0.772, d = 0.17), and 5Sit& Up (*p* = 0.001, d = 1.02) indicated the preliminary durability of the sensorimotor training effects over the short‐term follow‐up.

**Conclusions:**

The findings provide preliminary evidence that sensorimotor training may improve lower‐limb function, balance, gait, and joint proprioception in sedentary older women. Several motor and sensory outcomes showed notable gains, suggesting that this intervention could support postural control, walk efficiency, and potentially reduce fall risk in geriatric rehabilitation settings.

**Trial Registration:**

Iranian Registry of Clinical Trials (IRCT) No. IRCT20160815029373N8, Date: 04/07/2025

## 1 Introduction

With the increasing trend of aging worldwide and the prevalence of sensorimotor disorders, the decline in functional performance of the elderly has become a serious challenge in the healthcare field. Aging and the resulting physiological changes lead to decreased balance and postural control, increased risk of falls, decreased functional capabilities, and dependence on others in performing routine activities [1]. These changes are often further influenced by a sedentary lifestyle, leading to a decrease in quality of life and an increase in age‐related diseases [2]. Falls are more common in older people, especially those who are physically weaker and less active or who live in nursing homes. The consequences of falls can include soft tissue injuries, bone fractures, difficulties in performing routine activities, fear of falling, and even depression [3]. Improving balance is essential for fall prevention and requires efficient integration of sensory information from the visual, vestibular, and somatosensory systems. During movement, sensory neurons provide vital information about the position of limbs and their movement in space. Older adults rely more on visual information to maintain balance, which makes their balance more impaired in the absence of vision. Components of the proprioceptive system constitute an essential part of movement and play a key role in motor performance. Studies show that proprioceptive functions decline with age, which is associated with decreased balance and increased risk of falls [4, 5].

The use of fall prevention strategies plays an important role in reducing the incidence of falls and their consequences. Several studies have shown that training interventions, including balance training and gait training, are effective in reducing the risk of falls. Proprioceptive and sensorimotor training are among the promising approaches in preventing falls and improving balance [1, 6]. Sensorimotor training is widely recognized as an effective intervention for enhancing the integration of sensory input. These exercises primarily emphasize the use of proprioceptive signals, including kinesthetic information, particularly when visual feedback is reduced or absent, with the aim of improving and restoring sensorimotor function [7]. In lower‐limb proprioceptive programs, many studies incorporate walking on unstable surfaces, as this approach has been shown to improve motor responses and increase joint stability [6]. By increasing somatosensory and proprioceptive stimulation, sensorimotor exercises may also contribute to correcting muscle imbalances and promoting appropriate movement patterns at the level of the central nervous system [1]. Through the integration of sensory and motor information, the central nervous system improves the acquisition of skilled movements and enhances responsiveness to environmental perturbations, a process known as sensorimotor integration [8]. The interaction of the sensory and motor systems is a necessary condition for learning and performing skilled movements. The simultaneous activation of these two systems stimulates neuroplasticity at their junctions and contributes to the improvement of sensory and motor inputs to the sensorimotor integration centers. This is the process by which a person perceives and moves their body and the surrounding environment [9]. Researchers have found that exercises that focus on taking correct, fast, and purposeful steps can be very effective in preventing falls. Performing these exercises is especially appropriate on unstable surfaces, as it improves cognitive perception and sensorimotor abilities [10]. Moreover, recent cross‐sectional evidence suggests that proprioceptive function may be one of the strongest predictors of fall risk in older women [11]. This finding underscores the importance of interventions that specifically target proprioceptive feedback and sensorimotor integration to support balance and fall prevention in older adults.

Considering the increasing trend of aging in the world and its related functional consequences, effective interventions to improve balance, reduce the risk of falls, and enhance the quality of life of the elderly are strategic priorities in public healthcare systems. Evidence shows that reduced somatosensory perception and loss of sensorimotor integration are key factors in impaired balance and gait in the elderly [4, 5]. This problem is more severe in sedentary individuals and elderly residents of care centers due to the greater reduction in environmental stimuli and physical activities, and exposes them to frequent falls and their serious consequences. Meanwhile, functional sensorimotor training, emphasizing simultaneous stimulation of sensory and motor systems, have great potential for promoting neuroplasticity, improving movement patterns, and retraining balance [7, 10]. However, although some studies report potential benefits of these exercises, precise information based on gait kinematic data and evidence regarding the sustainability of their effects in sedentary older women have not yet been fully explored. Accordingly, the present study aimed to investigate the potential effects of functional sensorimotor‐based training on sedentary older adults, specifically to examine whether such training may improve gait and functional mobility, as measured by the Timed Up and Go test, walking time, and the lower limb strength, and to explore whether it may reduce fall‐related outcomes, including fear of falling and overall fall likelihood.

## 2 Methods

### 2.1 Study Design

The present study was a randomized clinical trial with a pre‐test, post‐test, and follow‐up design, including a control group. The study was conducted in accordance with the ethical standards of the Declaration of Helsinki and was approved by the Bioethics Committee of Guilan University (IR. GUILAN. REC.1403.175). The trial was registered in the Iranian Registry of Clinical Trials (IRCT No. IRCT20160815029373N8, registered on July 4, 2025). All participants were fully informed about the objectives, procedures, and potential risks of the study and provided written informed consent prior to participation. Participation was voluntary, and confidentiality of participant information was maintained throughout the study. This study was conducted and reported in accordance with the CONSORT (Consolidated Standards of Reporting Trials) guidelines, and the relevant CONSORT checklist has been completed.

The evaluation of research variables including balance, gait indices, joint position sense, and lower limb function was performed at three time points: pre‐test, post‐test (after 8 weeks), and follow‐up. During the pre‐test, participants performed an initial warm‐up to prevent injury. Following the pre‐test, the experimental group underwent a sensorimotor training protocol, while the control group continued their normal daily routine of general movements without receiving any additional exercise intervention.

### 2.2 Participants

**Figure 1:**
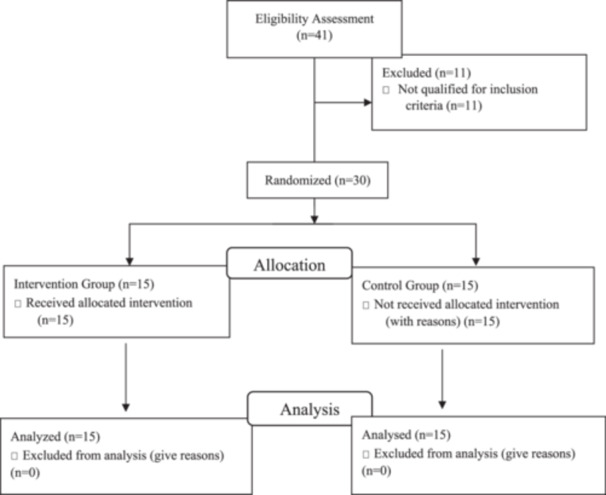
The study flowchart.

Participants were recruited from elderly care centers in Guilan Province, including the Rainbow Center, Zendegi Mana Center, and Rasht City Nursing Home. Recruitment was conducted through on‐site visits to these centers and coordination with the relevant administrators to identify and enroll eligible individuals. A total of 41 older women were initially screened for eligibility. Thirty eligible participants, all of whom had sedentary lifestyles, voluntarily enrolled in the study after providing written informed consent(Figure 1). To screen sedentary older adults, we adopted a definition widely used in previous research. According to Varo et al. (2003), a sedentary lifestyle is characterized by prolonged time spent in a sitting posture and minimal engagement in moderately to highly energetic leisure‐time activities (≥ 4 METs). In the present study, non‐participation in any structured exercise program during the past year was used as the primary screening criterion, while other low‐activity behaviors were qualitatively assessed to ensure a comprehensive determination of sedentary status [12]. Participants were randomly assigned to either the experimental group (*n* = 15), which received functional sensorimotor training, or the control group (*n* = 15) using a simple randomization method based on a list of names (odd numbers to the experimental group, even numbers to the control group). Group allocation was concealed from participants to maintain a single‐blind design. The required sample size was calculated using G*Power software, based on a 95% confidence level, 80% statistical power, and an effect size of 0.50 for the balance variable. For the repeated measures design, a minimum of 12 participants per group was required. To account for potential dropouts, 15 participants were allocated to each group [13, 14]. The appropriate sample size for each group was determined to be 14 individuals. Considering the probability of dropout, the final sample size was considered to be 30 individuals, and the participants were randomly assigned to two equal groups.

**Randomization:** Randomization was carried out by an independent researcher who was not involved in participant assessment or intervention delivery. Letters “A” and “B” were designated as indicators for group allocation and placed in opaque, sealed envelopes. The envelopes were then opened, and older adults were randomly assigned to one of the two groups. Consequently, participants were divided into a control group (A) and an FSMT intervention group (B). To minimize potential bias, group assignments remained concealed until participants were officially registered in the study, with access to allocation information restricted solely to the researcher responsible for managing the process. Furthermore, the study was conducted as a single‐blind trial: participants were unaware of their assigned group, while only the researchers responsible for delivering the intervention were informed of the group allocations.

### 2.3 Inclusion Criteria

The inclusion criteria for the study included sedentary elderly women aged 60 to 75 years [15]. They were residing in nursing homes in Gilan province and had no history of participating in regular exercise programs (at least two to 3 days per week) in the past year [12]. Participants had to have acceptable vision and hearing (with or without the use of hearing or visual aids), and in terms of personal performance, they had to be able to independently perform routine, personal, and health‐related activities. Also, their physical health was confirmed in terms of not having any diseases incompatible with the research exercise activities, including cardiovascular, respiratory, orthopedic, neurological, and infectious diseases, as well as the absence of acute or chronic lower limb injuries or surgery in that area in the past year (based on physician approval and medical record review). The ability to walk independently without the need for assistive devices and the ability to perform physical exercises were also essential. Cognitively, participants must have a score of 24 or higher on the Mini‐Mental State Examination (MMSE) [16, 17, 18] and be free of sedatives or other medications that affect cognitive function and balance.

### 2.4 Exclusion Criteria

Exclusion criteria included non‐participation in the pre‐test or post‐test; individual's unwillingness, family, or center officials to continue participating in the study, absence from more than two training sessions, recurrent dizziness during training, or any unusual injury or pain during or after training sessions (Figure 1).

### 2.5 Evaluation Tools and Methods

All assessments were conducted by a trained exercise science specialist. The assessor was not blinded to group allocation; however, all tests were performed using standardized procedures to ensure reliability and consistency of the measurements.

Cognitive status assessment: Mini‐Mental State Examination (MMSE): to assess the cognitive status of the participants, the Persian version of the MMSE test was used [19]. This 30‐point test includes the assessment of components such as orientation, memory, attention, calculation, language, and spatial‐structural abilities. A score of 24 or above was considered an indicator of cognitive health [17, 18, 20].

Timed Up and Go Test (TUG): to assess functional balance and fall risk, the TUG test was used. In this test, the time required to rise from a chair, walk 3 m, turn around, return, and sit down again was measured. A time greater than 13.5 s was considered the threshold for fall risk in the elderly [21].

Falls Efficacy Scale‐International (FES‐I): This scale has 16 items with a 4‐point Likert scale that assesses the level of stress of the elderly when performing daily activities. Higher scores indicate greater fear of falling and is a valid tool for assessing self‐efficacy and the level of psychological functioning related to balance [22].

10‐Meter Walk Test: Walking speed was assessed using a 10‐meter walking test, which included 2 m for acceleration and 2 m for deceleration. The time required to traverse the central 6 m was recorded, and walking speed was calculated in meters per second by dividing the 6‐meter distance by the elapsed time in seconds. Additionally, walking acceleration was determined by dividing the walking speed by the acceleration time. The test was conducted under two conditions: “comfortable” and “fast.” Notably, this test has been validated and shown to be reliable for older adults and is widely used to assess motor performance and walking ability in this population [23, 24].

Five Times Sit to Stand Test (FTSTS): the FTSTS test was used to assess lower limb strength, dynamic balance, and fall risk. Participants were asked to stand up from a sitting position on a chair and sit down again five times without using their hands. The mean time for the two repetitions was recorded as a performance index [25].

Assessment of knee joint position sense: to assess knee position sense, the active joint angle reconstruction test was used in a seated position, with an open motor chain, in a silent environment, and with blindfolds. After passively moving the knee to 45 degrees of extension, the participants were asked to actively repeat and maintain the movement. This assessment was performed three times for both legs, randomly [26]. For the ankle, the active joint angle reconstruction method was used; the participants were placed in a sitting position with a 90‐degree angle at the hip and knee joints. After passively inducing movement to a specified angle (20 degrees of plantar flexion or 10 degrees of dorsiflexion), the participants actively reconstructed and maintained the movement. Each test was repeated three times for each leg [27, 28].

### 2.6 Intervention: Functional Sensorimotor‐Based Training

The sensorimotor training program comprises a series of exercises designed to improve proprioception, neuromuscular coordination, balance, and motor control. The primary aim of the program was to refine proprioceptive processing, facilitate multisensory integration, and enhance dynamic postural stability in sedentary older adults. The intervention spanned 8 weeks, comprising 40‐min sessions delivered three times per week. All exercises adhered to the FITT principles (Frequency, Intensity, Time, and Type) and incorporated progressive overload to ensure sufficient challenge while prioritizing safety and injury prevention [1]. The exercises were conducted at the elderly care center in small groups of five participants to ensure safety and proper execution of the activities. Sessions were led by a specialist in older adult exercise, who had extensive experience in instructing and supervising sensorimotor and physical training for older adults.

Every training session was organized into three key phases. Warm‐up (5 min) and Main training (40 min): This phase included four progressively challenging categories of specialized exercises: Adhesive Star (enhancing proprioceptive control and movement coordination through diverse movement patterns), Colored Path (improving visuomotor perception and dynamic balance using structured, color‐coded pathways), Rubber Step (strengthening dynamic stability and static balance by adjusting platform height and incorporating dual‐task activities), and Obstacles on the Path (facilitating functional balance and adaptive motor responses by navigating obstacles and performing controlled triple flexion movements of the lower limbs), as shown in Table 1. Finally, the cool‐down (5 min) concluded the session [1].

**Table 1:** Progressive sensorimotor training program.

Week	Exercise	Intensity (Borg RPE)	Duration (time)	Exercise level
1–2	Warm‐up	Low intensity	5 min	
	Adhesive star (four‐point)	Moderate (5–6 out of 10)	7 min	Four‐point star with equal lengths
	Colored path ‐ Level 1	Moderate (5–6 out of 10)	7 min	Follow one color, return using the same color
	Rubber step ‐ Level 1	Moderate (5–6 out of 10)	7 min	5 cm step height, throwing a light ball to the examiner
	Obstacles on the path ‐ Level 1	Moderate (5–6 out of 10)	7 min	Three obstacles (H: 5 cm, W: 8 cm, L: 40 cm)
	Cool‐down	Low intensity	5 min	
3–4	Warm‐up	Low intensity	5 min	
	Adhesive star (four‐point)	Moderate (6 out of 10)	7 min	Four‐point star with different lengths
	Colored path ‐ Level 2	Moderate (6 out of 10)	7 min	Follow one color, return using a different color
	Rubber step ‐ Level 2	Moderate (6 out of 10)	7 min	Step up and down before throwing the ball
	Obstacles on the path ‐ Level 2	Moderate (6 out of 10)	7 min	Three obstacles (H: 10 cm, W: 8 cm, L: 40 cm)
	Cool‐down	Low intensity	5 min	—
5–6	Warm‐up	Low intensity	5 min	—
	Adhesive star (eight‐point)	Moderate (6 out of 10)	7 min	Eight‐point star with equal lengths
	Colored path ‐ Level 3	Moderate (6 out of 10)	7 min	Follow alternating colors and return using alternating colors
	Rubber step ‐ Level 3	Moderate (6 out of 10)	7 min	10 cm step height, step up and down before throwing the ball
	Obstacles on the path ‐ Level 3	Moderate (6 out of 10)	7 min	Three cones (blue, green, white) randomly placed
	Cool‐down	Low intensity	5 min	—
7–8	Warm‐up	Low intensity	5 min	—
	Adhesive star (eight‐point)	Moderate (7 out of 10)	7 min	Eight‐point star with different lengths
	Colored path ‐ Level 4	Moderate (7 out of 10)	7 min	Follow colors based on verbal instructions
	Rubber step ‐ Level 4	Moderate (7 out of 10)	7 min	10 cm step height, alternating commands
	Obstacles on the path ‐ Level 4	Moderate (7 out of 10)	7 min	Combination of all obstacles in random order
	Cool‐down	Low intensity	5 min	—

To ensure proper execution of movements and participant safety, a minimum of two introductory sessions were conducted prior to the full program. Perceived exercise intensity was monitored for each exercise using the Borg RPE scale, with participants reporting their level of effort at the end of each exercise. When intensity fell below the target range (moderate intensity, RPE 5–7/10 depending on exercise level and week), the trainer individualized the exercises by adjusting task complexity, resistance, movement speed, or incorporating additional challenge elements within the same exercise category.

Exercises were gradually progressed in both complexity and intensity over the 8‐week program to enhance motor function, support neuromuscular adaptation, and reduce fall risk. The training targeted neural pathways involved in balance coordination, regulated sensorimotor input, and activated mechanoreceptors, while simultaneously optimizing postural control and dynamic stability. This structured approach provided a safe and effective environment for improving physical performance and promoting greater independence in movement among older adults. A few participants reported mild and transient muscle soreness during the initial sessions, which resolved spontaneously without any intervention. Throughout the intervention, exercise adherence, attendance rates, and adverse events were closely monitored and recorded. During the 2‐month follow‐up period, participants' motor performance was monitored through telephone check‐ins and in‐person visits. These contacts were solely for assessment purposes, and no additional intervention, training sessions, or exercises were provided during this period.

### 2.7 Statistical Analysis

in the present study, descriptive statistics, including mean and standard deviation, were used to describe the demographic variables of the sample (e.g., age, height, weight, etc.). The Shapiro‐Wilk test was applied to assess the normality of data distribution, and Levene's test was used to ensure the homogeneity of variances. To examine the effect of time (pre‐test, post‐test, and follow‐up) and group (different training groups and control group) on the studied variables, 2 × 3 repeated measures analysis of variance (RMANOVA) was conducted. Bonferroni post‐hoc tests were performed for pairwise comparisons between different time points. The training programs' effect sizes on the dependent variables were categorized as small (0.01–0.2), medium (0.2–0.5), and large ( ≥ 0.8) using Cohen's d. The effect size between groups was also determined using Partial Eta Squared (η²), which was divided into three categories: small (≤ 0.01), medium ( ≤ 0.06), and large ( ≥ 0.14). IBM SPSS software (version 25) was used for all statistical analyses, which were carried out at a significance level of *p* < 0.01.

## 3 Results

The results are presented in two sections: descriptive and inferential statistics. Descriptive statistics for demographic characteristics (age, height, weight, body mass index, and MMSE scores), along with the results of the baseline homogeneity assessment, are reported in Table 2. All participants completed the 8‐week intervention, and attendance rates exceeded 95% across all participants. No adverse events were reported during the intervention period.

**Table 2:** The demographic characteristics of the participants (mean ± standard deviation).

Measurement index	Groups	Mean ± SD	T	*p*
Age (yr)	Control	73.53 ± 4.53	0.57	0.56
	Experimental	70.87 ± 4.53		
Height (cm)	Control	153.53 ± 6.05	0.68	0.43
	Experimental	151.27 ± 9.02		
Weight (kg)	Control	63.63 ± 8.86	1.14	0.35
	Experimental	63.02 ± 12.57		
BMI (kg/m^2^)	Control	26.95 ± 2.95	0.86	0.38
	Experimental	27.38 ± 4.35		
MMSE	Control	25.66 ± 1.98	1.12	0.29
	Experimental	26.06 ± 1.75		

Abbreviations: cm, centimeter; kg, kilogram; SD, standard deviation; yr, year.

As observed in Table 2, the results of the independent t‐test indicated that there were no significant differences between the two groups regarding age, height, weight, body mass index, and MMSE (*p* > 0.01), suggesting that the two groups are homogeneous in all of the aforementioned aspects. Shapiro‐Wilk test was used to assess and confirm the normality of the data distribution

TW: Time of Walking; VW: Velocity of Walking; AW: Acceleration of Walking; FOF Score: Fear of falling; TUG Score: Time Up & Go; JPS: Joint Position Sense; R: Right; L: Left; F: Flexion; G: Group;E: Experimental; C: Control; Pre‐t: Pre‐test; Post‐t: Post‐test; F‐up: Follow‐up; M ± SD: Mean ± Standard Deviation; G×T Effect: Group × Time Effect; η² = Partial Eta Squared (Effect size: Small Effect: 0.01, Medium Effect: 0.06, Large Effect: 0.14), *Significance: *p* < 0.01

Table 3 summarizes the repeated measures ANOVA results for all outcome variables across three assessment points (pre‐test, post‐test, and follow‐up) and between groups. In addition to statistical significance, partial eta squared (η²) is reported to quantify effect size.

**Table 3:** Repeated measures ANOVA (multiple variables).

Variables	G	Pre‐t	Post‐t	F‐up	Time effect			Group effect			G × T effect		
		(M ± SD)	(M ± SD)	(M ± SD)	(F)	(*p*)	(η²)	(F)	(*p*)	(η²)	(F)	(*p*)	(η²)
FOF Score	C	50.53 ± 10.99	50.60 ± 11.46	50.80 ± 11.53	0.107	0.899	0.008	9.066	< 0.001*	0.405	0.132	0.719	0.005
	E	53.27 ± 5.55	46.47 ± 5.55	48.73 ± 5.65	18.152	< 0.001*	0.573						
TUG Score	C	16.92 ± 5.86	15.50 ± 6.31	17.17 ± 6.06	12.486	< 0.001*	0.480	23.495	< 0.001*	0.456	1.986	0.170	0.066
	E	16.71 ± 1.99	11.43 ± 1.71	14.58 ± 2.44	144.94	< 0.001*	0.915						
TW (s)	C	14.12 ± 3.94	14.3 6 ± 4.08	13.96 ± 4.19	0.73	0.491	0.051	89.42	< 0.001*	0.86	4.270	0.048	0.132
	E	13.19 ± 2.97	8.81 ± 2.92	12.63 ± 3.65	157.81	< 0.001*	0.921						
VW (m/s)	C	0.75 ± 0.19	0.74 ± 0.19	0.76 ± 0.19	0.205	0.816	0.015	42.070	< 0.001*	0.757	7.520	0.011	0.212
	E	0.78 ± 0.14	1.21 ± 0.29	0.82 ± 0.17	77.326	< 0.001*	0.851						
AW (m/s^2^)	C	0.122 ± 0.06	0.117 ± 0.06	0.124 ± 0.06	0.102	0.904	0.007	25.539	< 0.001*	0.654	9.051	0.006*	0.244
	E	0.128 ± 0.04	0.313 ± 0.13	0.142 ± 0.25	47.560	< 0.001*	0.779						
5Sit& up	C	17.87 ± 3.20	18.60 ± 3.29	17.60 ± 2.87	4.384	0.022	0.245	22.118	< 0.001*	0.621	17.153	< 0.001*	0.380
	E	15.33 ± 3.01	12.33 ± 2.55	13.60 ± 2.47	36.829	< 0.001*	0.732						
R‐dorsi‐F (JPS)	C	4.46 ± 1.02	4.56 ± 0.90	4.58 ± 0.93	0.559	0.051	0.578	8.137	0.002*	0.376	4.891	0.035	0.149
	E	3.86 ± 1.92	3.16 ± 1.28	3.53 ± 1.39	16.622	< 0.001*	0.552						
l‐dorsi‐F (JPS)	C	4.65 ± 1.23	4.61 ± 1.13	4.91 ± 0.90	8.144	0.002*	0.376	8.353	0.002*	0.382	10.948	0.003*	0.281
	E	3.78 ± 1.30	3.08 ± 0.99	3.46 ± 0.87	27.970	< 0.001*	0.674						
R‐plantar‐F (JPS)	C	3.26 ± 0.75	3.36 ± 0.69	3.45 ± 0.62	1.683	0.111	0.205	19.655	< 0.001*	0.593	0.073	0.789	0.003
	E	3.74 ± 1.32	3.14 ± 0.97	3.45 ± 0.87	41.057	< 0.001*	0.753						
l‐plantar‐F (JPS)	C	3.42 ± 0.63	3.40 ± 0.78	3.60 ± 0.70	6.885	0.004*	0.338	7.529	0.003*	0.358	0.150	0.701	0.005
	E	3.87 ± 1.23	3.34 ± 0.78	3.57 ± 0.74	17.279	< 0.001*	0.561						
R‐knee‐F45 (JPS)	C	4.51 ± 1.18	4.64 ± 1.09	4.64 ± 0.97	0.450	0.642	0.032	8.193	0.002*	0.378	2.925	0.098	0.095
	E	4.28 ± 1.76	3.35 ± 1.11	3.80 ± 1.52	13.173	< 0.001*	0.494						
l‐knee‐F45 (JPS)	C	3.98 ± 1.50	4.00 ± 1.5	4.18 ± 1.25	1.975	0.158	0.128	8.864	< 0.001*	0.396	1.000	0.326	0.034
	E	4.00 ± 115	3.25 ± 0.76	3.63 ± 0.72	19.248	< 0.001*	0.588						

* Significance: *p* < 0.01.

A significant main effect of time was observed for several outcomes, indicating overall changes across the measurement occasions. Significant group effects were also found for most variables, suggesting overall differences between the experimental and control groups. Notably, large between‐group effect sizes were observed for walking‐related measures, including TW (η² = 0.860), VW (η² = 0.757), and AW (η² = 0.654).

Importantly, significant Group × Time interactions were identified for TW (*p* = 0.048), VW (*p* = 0.011), AW (*p* = 0.006), and 5Sit& Up (*p* < 0.001), as well as several JPS outcomes. These interactions indicate that the pattern and magnitude of change over time differed between groups, supporting an intervention‐related effect for these variables. Interaction effect sizes ranged from medium to large, with notable values for AW (η² = 0.244), VW (η² = 0.212), and 5Sit& Up (η² = 0.380).

For FOF and TUG, the Group × Time interaction was not statistically significant (*p* = 0.719 and *p* = 0.170, respectively), indicating that differential changes between groups could not be confirmed for these outcomes based on the ANOVA model alone. Post hoc pairwise comparisons among time points are presented in Table 4 using Bonferroni adjustment.

**Table 4:** Bonferroni‐adjusted pairwise comparisons across pre‐test, post‐test, and follow‐up within study groups.

Variable	Comparison	Control			Experimental		
		Mean difference	*p*‐value	Effect size (d)	Mean difference	*p*‐value	Effect size (d)
FOF Score	Pre vs post	−0.070	0.821	0.03	−6.800	0.003*	0.34
	Pre vs follow	0.270	0.643	0.05	−4.540	0.012	0.28
	Post vs follow	0.200	0.715	0.04	2.260	0.048	0.16
TUG Score	Pre vs post	1.419	0.001	0.25	5.286	< 0.001*	2.45
	Pre vs follow	−0.250	1.000	−0.05	2.133	0.001*	1.22
	Post vs follow	−1.669	0.003	−0.27	−3.153	< 0.00*	1.43
TW	Pre vs post	−0.239	1.000	−0.06	4.379	< 0.00*	1.49
	Pre vs follow	0.158	1.000	0.04	0.559	0.772	0.17
	Post vs follow	0.397	1.000	0.10	−3.819	0.001*	−1.16
VW	Pre vs post	0.013	1.000	0.05	−0.431	0.001*	−1.89
	Pre vs follow	−0.010	1.000	−0.05	−0.040	0.686	−0.26
	Post vs follow	−0.023	1.000	−0.11	0.391	0.001*	1.64
AW	Pre vs post	0.004	1.000	0.08	−0.185	0.001*	−1.92
	Pre vs follow	−0.003	1.000	−0.03	−0.014	0.639	−0.08
	Post vs follow	−0.007	1.000	−0.12	0.171	0.001*	0.86
5Sit& Up	Pre vs post	−0.733	0.231	−0.22	3.000	< 0.00*	1.56
	Pre vs follow	0.267	0.757	0.09	1.733	0.001*	1.02
	Post vs follow	1.000	0.023	0.32	−1.267	0.003*	−0.89
R‐dorsi‐F(JPS)	Pre vs post	−0.093	1.000	−0.05	0.700	0.001*	0.95
	Pre vs follow	−0.120	1.000	−0.06	0.327	0.062	0.42
	Post vs follow	−0.027	1.000	−0.03	−0.373	0.001*	−0.48
l‐dorsi‐F(JPS)	Pre vs post	0.040	1.000	0.03	0.693	0.001*	0.92
	Pre vs follow	−0.260	0.136	−0.20	0.313	0.053	0.38
	Post vs follow	−0.300	0.001*	−0.25	−0.380	0.001*	−0.50
R‐plantar‐F(JPS)	Pre vs post	−0.100	0.904	−0.07	0.607	< 0.00*	0.89
	Pre vs follow	−0.193	0.331	−0.12	0.300	0.048	0.41
	Post vs follow	−0.093	0.306	−0.09	−0.307	0.001*	−0.45
l‐plantar‐F(JPS)	Pre vs post	0.020	1.000	0.02	0.527	< 0.00*	0.80
	Pre vs follow	−0.180	0.212	−0.15	0.333	0.005*	0.47
	Post vs follow	−0.200	0.003*	−0.22	−0.193	0.004*	−0.35
R‐knee‐F45(JPS)	Pre vs post	0.013	1.000	0.63	−0.431	< 0.00*	0.77
	Pre vs follow	−0.010	1.000	0.29	−0.040	0.686	0.39
	Post vs follow	0.023	1.000	−0.34	0.391	0.001*	−0.51

Abbreviations: AW, Acceleration of Walking; C, Control; E, Experimental; F, Flexion; FOF Score, Fear of falling; L, Left; R, Right; TUG Score, Time Up & Go; TW, Time of Walking; VW, Velocity of Walking.

*Significance: *p* < 0.01; Effect Size d: Cohen's d (Effect size: Small less than 0.2, Medium between 0.5 and 0.8, Large 0.8).

Sensorimotor training led to significant improvements in functional performance and balance among older adults. In the experimental group, fear of falling (FES‐I) significantly decreased from pre‐ to post‐test (*p* = 0.003, ES = 0.34) and was partially maintained at follow‐up (*p* = 0.048, ES = 0.16). Functional balance, assessed using the Timed Up and Go (TUG), showed substantial improvement (*p* = 0.001, ES = 2.45), which remained significant at follow‐up (*p* = 0.001, ES = 1.22). Notably, due to the calculation of mean differences as Pre minus Post, some effect sizes—for example, walking velocity and acceleration—appear negative in the table, although actual performance increased. Overall, these results indicate that the sensorimotor training program meaningfully enhanced motor function and outcomes related to fall risk.

The post‐test showed a significant improvement in walking speed and acceleration (*p* = 0.001, ES = 1.89 and *p* = 0.001, ES = 1.92, respectively), while the follow‐up assessment indicated a partial decline toward baseline levels (*p* = 0.001, ES = 1.64 and *p* = 0.001, ES = 0.86, respectively), suggesting that continued practice may be necessary to maintain these gains. Sit‐to‐stand performance (5Sit& Up) in the experimental group improved significantly from pre‐ to post‐test (*p* = 0.001, ES = 1.56) and was maintained at follow‐up (*p* = 0.001, ES = 1.02). Ankle and knee proprioception (R‐/l‐dorsi‐F, R‐/l‐plantar‐F, R‐/l‐knee‐F45) also showed significant improvements post‐intervention (*p* = 0.001, ES ≈ 0.8–0.95) with partial retention at follow‐up (*p* = 0.001, ES ≈ −0.45 to −0.50). In contrast, the control group showed no significant changes in any of the variables (*p* > 0.05, ES < 0.3). These findings indicate that sensorimotor training effectively enhances motor performance, balance, and proprioceptive function in older adults, and continued training is recommended to maintain these benefits.

## 4 Discussion

The results of the present study demonstrated that functional sensorimotor‐based training in conjunction with dynamic walking challenges was associated with improvements in various aspects of balance, proprioception, and motor function. One of the findings of this intervention was a reduction in fear of falling among participants who underwent this training program. This reduction was accompanied by an improvement in proprioceptive awareness, suggesting that performing activities with an unpredictable nature, such as overcoming obstacles, walking on uneven surfaces, and responding to environmental changes, may contribute to enhanced postural control during daily activities. Although these improvements were partially retained at follow‐up, slight declines over time highlight the potential importance of continuous challenge or stimulation of the sensorimotor system to maintain the gains achieved by these exercises.

Likewise, in the area of functional balance, the intervention group showed improvements, especially during a test that required rapid and predictable adjustments, such as the Timed Up and Go test. These findings could reflect preliminary improvements in sensorimotor integration and neuromuscular coordination, which might be related to exposure to conditions with changes in speed, direction of movement, and multisensory input. The persistence of these results at follow‐up may indicate that this type of training protocol could support adaptations in neural pathways involved in maintaining balanced posture under different motor conditions. The adaptations observed in gait may also be indicative of positive intervention effects. The cognitive load of learning the walking test during the early stages of motor adaptation may have contributed to participants' higher walking test scores in the early stages by encouraging conscious and systematic walking patterns. However, despite significant improvements in walking speed and acceleration at post‐test, a partial regression toward baseline was observed at follow‐up. This finding suggests that continued regular practice is essential to maintain the intervention effects on motor performance. Similarly, improvements in transitional movements could be associated with better performance in the sit‐to‐stand test, an indicator of lower limb function in older adults, which may support the idea that training focused on dynamic body movements can contribute to improved muscle coordination and postural control [29, 30]. This type of training seems to result in timely muscle activation and increased involvement of proprioception, leading to smoother functional movements [31]. With a slight decrease in proprioception accuracy reported during follow‐up, significant improvements were still observed in ankle and knee proprioception function, but with partial retention of the effect. These initial improvements may reflect the adaptive capacity of the sensorimotor system in response to goal‐oriented and task‐oriented challenges, while reductions at follow‐up could suggest the need for longer or continuous training to sustain these effects.

Based on the findings of the present study, previous research has shown that interventions such as balance training and gait training may improve balance and reduce the risk of falls [13, 32, 33, 34]. Proprioceptive and sensorimotor training have been reported as potentially effective interventions for preventing falls and enhancing balance [8, 14, 35]. Moreover, the results of this study may have been influenced by the implementation of the intervention in elderly care centers under the supervision of a trained specialist. This setting facilitated higher participant engagement, consistent attendance, and the absence of dropouts. Therefore, the findings likely reflect the true effects of the intervention in a structured environment with professional support.

Although the proprioceptive system is the essence of movement and plays a key role in individuals' motor control, the ability to maintain balance depends on the effective integration of sensory information received from the visual, vestibular, and proprioceptive systems. During movement, the somatosensory system provides vital information about the position and movement of limbs in space [9]. However, older adults people usually rely more on visual information to maintain balance, which causes a significant decrease in balance, especially in conditions where vision is impaired [36]. On the other hand, proprioceptive function also decreases with age, which is associated with difficulty in maintaining balance and an increased risk of falling [9, 36]. In this regard, we can mention the study by Ahmad et al., who investigated the effect of sensorimotor training on improving the reception of somatosensory and proprioceptive stimuli, reducing muscle imbalances, and creating a precise movement pattern in the central nervous system [8]. In our study, sensorimotor training combined with dynamic walking challenges resulted in significant improvements in variables such as fear of falling (FOF), time to get up and go (TUG), walking speed (TW), and joint position sense at the wrist and knee. These changes, observed over the course of the study (pretest, posttest, and follow‐up), may indicate positive effects of sensorimotor training on motor performance and confidence, although causality cannot be fully established. Fear of falling (FOF) decreased from 53.27 at baseline to 46.47 post‐intervention, representing a statistically significant improvement. At the 2‐month follow‐up, the mean FOF slightly increased to 48.73 but remained below baseline levels. Although no universally accepted minimal clinically important difference (MCID) has been established for the FES‐I, this reduction may reflect meaningful clinical benefits in daily function and confidence in mobility. The slight increase at follow‐up suggests that ongoing practice or maintenance sessions may be necessary to sustain the clinical effects.

Previous meta‐analyses have explored how exercise interventions can improve balance and mobility in older adults. Zhang et al. (2026) reported that task‐oriented training appeared to offer meaningful benefits, showing moderate to large effects on balance (BBS: MD = 2.58), moderate effects on mobility (TUG: MD = −0.55), and small to moderate improvements in gait speed. Similarly, Yu et al. (2025) found relatively large effects on fall efficacy (Hedges' g = 1.01), moderate to large effects on balance function (g = 0.89), and moderate improvements in TUG performance following exercise interventions [37, 38].

In our study, participants in the experimental group demonstrated generally large to very large effect sizes across the main functional outcomes. Very large effects were observed for TUG (η² = 0.915) and 10MWT (η² = 0.921), while a large effect was seen for FTSTS (η² = 0.732), with notable improvements also in FES‐I scores. Overall, these effect sizes appear broadly consistent with and in some functional measures slightly exceed the pooled moderate to large effects reported in previous meta‐analyses, suggesting that the intervention may have had a relatively strong impact within the context of existing evidence [37, 38].

Previous studies have suggested that an 8‐week combined exercise program, including balance training, multi‐directional movements, and lower‐limb strengthening, may help reduce fall risk in older women by improving balance, muscle strength, proprioception, and postural stability [39]. These findings are broadly consistent with the results of the present study, as our sensorimotor‐based training, which incorporated dynamic walking challenges and functional activities, was associated with relative improvements in balance, neuromuscular coordination, and motor performance. Together, these observations underscore the potential value of multicomponent training for supporting sensorimotor function and reducing fall risk in older adults, while acknowledging that further research is needed to confirm these effects.

Sensorimotor training specifically focuses on proprioceptive signals such as kinesthetic (movement) and tactile messages, especially in situations where sensory inputs such as vision are reduced or absent. The ultimate goal of these exercises is to improve and restore sensorimotor functions. Many studies have used walking exercises on uneven surfaces to enhance lower limb proprioception, improve motor responses, and increase joint stability [9, 35]. A study by Da Silva et al. also used rotational training to improve sensorimotor function, which is the same approach used in our study. They performed exercises on stable and unstable surfaces, in which participants improved their motor and cognitive skills using rotational training under different conditions [40]. Morat etal. emphasized that sensorimotor gait training may be more effective than traditional balance training in improving balance and preventing falls [41]. In the present study, walking training in different directions was associated with improvements in functional balance and a reduced risk of falls. Exercises on unstable surfaces could have contributed to improved proprioception, muscle coordination, and motor strategies to maintain balance during daily activities. Walking speed (TW) and walking velocity (VW) improved in the experimental group, consistent with findings from Da Silva et al [40, 42]. Therefore, sensorimotor training may be considered a promising intervention for supporting motor performance and fall prevention, although further research is needed to confirm these effects [9, 40, 43, 44].

These results, together with other scientific evidence, suggest potential benefits of sensorimotor training for improving motor function, balance, and proprioception, particularly in older adults and those at higher risk of falls. Overall, the findings of this study indicate that sensorimotor functional training combined with sensorimotor exercises may preliminarily enhance motor function, balance, joint position sense, and possibly reduce fear of falling in older adults. Based on these observations, similar training programs aimed at strengthening the sensorimotor system could be implemented for older adults and individuals with movement disorders to support positive outcomes and potentially help prevent injuries related to imbalance and falls.

These results, together with other scientific evidence, suggest the potential benefits of sensorimotor training for improving motor function, enhancing balance, and supporting proprioception, particularly in older adults and those at higher risk of falls. Overall, the findings of this study indicate that sensorimotor functional training combined with sensorimotor exercises may preliminarily enhance motor function, balance, joint position sense, and possibly reduce fear of falling in older adults. Based on these observations, it is recommended that similar training programs aimed at strengthening the sensorimotor system be implemented on an ongoing basis for older adults and individuals with movement disorders to support these positive outcomes and potentially help prevent injuries related to imbalance and falls.

Importantly, the present findings highlight the potential novelty and advantages of sensorimotor‐based training compared with conventional balance and gait interventions. Unlike standard programs that primarily involve repetitive balance tasks, the sensorimotor training in this study incorporated dynamic walking challenges, multi‐directional movements, and multi‐sensory stimulation, specifically targeting neuromuscular coordination, proprioceptive feedback, and adaptive postural control [8, 45]. This combination of unpredictable, functional tasks appears to promote not only improvements in static and dynamic balance, but also enhanced gait efficiency, reduced fear of falling, and better overall functional mobility [46, 47].

Limitations: While this study provides strong evidence of how sensorimotor training within 8 weeks can improve balance, motor function, and reduce fear of falling, it has a number of limitations that should be considered when interpreting the results. The study sample consisted of older adults' residents with limited mobility who lived in nursing homes. Although this selection of participants was intended to control for confounding variables, it may limit the applicability of the findings to other populations, such as older adults' men, more active seniors, or individuals living in the community. As a result, caution is needed when extrapolating these results to broader, more diverse populations. The follow‐up period was relatively short, and the results were measured only a short time after the intervention. Consequently, this limits the ability to draw conclusions regarding the long‐term sustainability of the observed effects. Despite the use of functional performance tests, other important psychological factors such as baseline activity levels, movement confidence, and personal motivation were not quantitatively assessed. Due to equipment constraints, more advanced techniques, such as movement analysis and electromyography, could not be used to accurately examine muscle changes and gait cycles.

## 5 Conclusion

The results of this study provide preliminary evidence that sensorimotor and functional training programs may contribute to improvements in balance, gait, proprioception, and reduction of fear of falling among older adults. The slight decline in some outcomes during the short‐term follow‐up suggests that, while many adaptations are maintained, longer‐term interventions and follow‐up are needed to achieve more durable benefits. Future research is recommended to explore modifications in exercise content, frequency, duration, and progression to optimize retention and performance in older populations. Implementing such programs in controlled environments under the guidance of trained instructors may help older adults, particularly residents of nursing homes, to maintain individual independence and functional capacity. Further studies should also evaluate the program in more diverse and realistic settings to examine its effectiveness in less controlled environments. Finally, longer‐term follow‐up studies are necessary to fully assess the sustained effects of sensorimotor training.

## Author Contributions

**Parisa Sedaghati:** funding acquisition, conceptualization, methodology, data curation, supervision, formal analysis, validation, writing – original draft, writing – review and editing, project administration, investigation, resources, visualization. **Fatemehzahra Talebi kenari:** formal analysis, validation, writing – original draft, writing – review and editing, investigation, resources, visualization.

## Ethics Statement

All procedures were approved by the Ethics Committee of Guilan University (IR. GUILAN. REC.1403.175), whose guidelines are by the Declarations of Helsinki. All participants entered the study after completing and signing the Informed Consent Form and the study was confirmed by a supervisor appointed.

## Conflicts of Interest

The authors declare no conflicts of interest.

## 1 Data Availability Statement

The data that support the findings of this study are available on request from the corresponding author. The data are not publicly available due to privacy or ethical restrictions.

## Transparency Statement

The lead author Parisa Sedaghati affirms that this manuscript is an honest, accurate, and transparent account of the study being reported; that no important aspects of the study have been omitted; and that any discrepancies from the study as planned (and, if relevant, registered) have been explained.
